# Effects of intermittent (5:2) or continuous energy restriction on basal and postprandial metabolism: a randomised study in normal-weight, young participants

**DOI:** 10.1038/s41430-021-00909-2

**Published:** 2021-05-26

**Authors:** Yangfan Gao, Kostas Tsintzas, Ian A. Macdonald, Sally M. Cordon, Moira A. Taylor

**Affiliations:** 1 Division of Physiology, Pharmacology and Neuroscience, School of Life Sciences, University of Nottingham, Queen’s Medical Centre, Nottingham, UK; 2 National Institute for Health Research (NIHR) Nottingham Biomedical Research Centre, Nottingham, UK; 3 MRC Versus Arthritis Centre for Musculoskeletal Ageing Research, Nottingham, UK

**Keywords:** Randomized controlled trials, Weight management

## Abstract

**Background/objectives:**

Intermittent energy restriction (IER) may overcome poor long-term adherence with continuous energy restriction (CER), for weight reduction. We compared the effects of IER with CER for fasting and postprandial metabolism and appetite in metabolically healthy participants, in whom excess weight would not confound intrinsic metabolic differences.

**Subjects/methods:**

In a 2-week randomised, parallel trial, 16 young, healthy-weight participants were assigned to either CER (20% below estimated energy requirements (EER)) or 5:2 IER (70% below EER on 2 non-consecutive days; 5 days at EER, per week). Metabolic and appetite regulation markers were assessed before and for 3 h after a liquid breakfast; followed by an ad libitum lunch; pre- and post-intervention.

**Results:**

Weight loss was similar in both groups: −2.5 (95% CI, −3.4, −1.6) kg for 5:2 IER vs. −2.3 (−2.9, −1.7) kg for CER. There were no differences between groups for postprandial incremental area under the curve for serum insulin, blood glucose or subjective appetite ratings. Compared with CER, 5:2 IER led to a reduction in fasting blood glucose concentrations (treatment-by-time interaction, *P* = 0.018, *η*^2^_*p*_ = 0.14). Similarly, compared with CER, there were beneficial changes in fasting composite appetite scores after 5:2 IER (treatment-by-time interaction, *P* = 0.0003, *η*^2^_*p*_ = 0.35).

**Conclusions:**

There were no significant differences in postprandial insulinaemic, glycaemic or appetite responses between treatments. However, 5:2 IER resulted in greater improvements in fasting blood glucose, and beneficial changes in fasting subjective appetite ratings.

## Introduction

Obesity increases the risk of cardiovascular diseases [1], T2DM [2], dyslipidaemia [3], dementia [4], cancers [5] and premature mortality [6]. Body weight (BW) reduction of 5–10% is recommended to improve metabolic function [7], using continuous energy restriction (CER) [8]. However, metabolic adaptations in appetite and energy utilisation [9–12] and poor compliance [13–16] limit success. 5:2 intermittent energy restriction (5:2 IER) is a popular alternative involving 2 days of energy restriction per week interspersed with unrestricted, or less restricted periods [17].

In people with overweight or obesity, compared with CER, 5:2 IER, with 2, non-consecutive energy-restricted days/week, resulted in comparable reductions in BW, fat mass and fat-free mass, and improved glycated Hb levels and fasting metabolic parameters, over 3, 6 or 12 months [18–22]. Energy restriction on 2 consecutive days/week, compared with CER, has demonstrated greater reductions in fasting insulin concentration, insulin resistance (homoeostasis model assessment of insulin resistance, HOMA-IR) and fat mass over 3 or 6 months, with comparable energy deficits and weight loss [16, 23]. As a consequence of modern lifestyles, potentially much of the 24-h period is typified by an absorptive state; however, there are limited data on the impact of 5:2 IER on postprandial metabolism. In one study, postprandial TAG decreased with 5:2 IER but increased with CER [24]. Differences were attributed to the shortened absorptive period during the 2 consecutive energy-restricted days.

This study thus aims to compare both the fasting and postprandial metabolic and appetitive effects of 2 weeks of 5:2 IER (with non-consecutive energy-restricted days) and CER. Participants were healthy, young, and of normal weight to minimise the potential impact on treatment differences, of metabolic defects associated with overweight and obesity. All food and beverages were provided to improve compliance. The main objective of this study was to compare the incremental area under the curve (iAUC) for serum insulin, concurrently with other metabolic and appetite responses, for 3 h after a standardised liquid breakfast following 2 weeks of CER and 5:2 IER.

## Methods

### Participants

Sixteen healthy men and women (20–35 years, BMI 20–25 kg/m^2^, waist circumference < 94 cm for men and <80 cm for women) were recruited from the University of Nottingham and Queen’s Medical Centre. They were moderately physically active (International Physical Activity Questionnaire (IPAQ)) [25] with a stable weight (±2 kg) over the previous 3 months. Exclusion criteria are shown in Supplementary Information 1. Participants attended five visits (1 × 10 min, 1 × 30 min, 1 × 1 h and 2 × 6 h) from November 2018 to May 2019 (Supplementary Fig. 1). The study was approved by the University of Nottingham, Faculty of Medicine and Health Sciences Research Ethics Committee (Reference Number 121-1809), conformed to the World Medical Association Declaration of Helsinki and was registered at ClinicalTrials.gov PRS: NCT04138160.

### Screening

BW, height, waist and hip circumferences, heart rate (HR), systolic and diastolic blood pressure (BP) were measured and a blood sample obtained for routine investigation. Skinfold thicknesses (four sites) were measured to estimate body fat percentage [26, 27]. A Personal Information and General Health form, the Beck Depression Inventory [28], the Eating Attitudes Test-26 [29] and the IPAQ were completed.

### Study design

A randomised, parallel-armed design compared 5:2 IER with CER, for 2 weeks. With 5:2 IER, food was provided for 5 days to meet the participants’ estimated energy requirements (EER) and for 2 non-consecutive days as a 70% energy-restricted diet. For the CER, food was restricted on all days (20% energy restriction), resulting in the same overall energy restriction in the two diets. Normal dietary and physical activity patterns were maintained during a 1-week, pre-study period commencing at the beginning of the 2nd week of the menstrual follicular phase, in women. Waking free-living physical activity level (PAL, daily step counts) was estimated using a pedometer (Omron Walking Style One 2.1, Omron, USA). Following a pre-intervention laboratory visit and a 7-day gap (to ensure post-intervention measurements were also conducted during the 2nd week of the follicular phase for women) the experimenter randomly assigned the participants to a treatment group using computer-generated randomisation (https://www.randomizer.org/) and the intervention period began. On Day 7 of each 2-week intervention, participants attended the laboratory in the morning, fasting, for BW assessment and subcutaneous continuous glucose monitoring device (CGM) attachment. Glucose values were reviewed across 24 h, the day hours and the night hours, for the mean, maximum, minimum, iAUC, SD and %CV. Whole-day glycaemic profile was plotted by calculating the mean of each of the 6 days of measurement (Days 8–13) at a timepoint, for a participant, and then calculating the mean at the timepoint for the six participants in each treatment group.

### Dietary intervention periods

Energy provision was based on estimated basal metabolic rate [30] and daily PAL [31, 32] adjusted for the deficit required. The 70% energy restriction applied on Days 1, 4, 8 and 11 of 5:2 IER (Supplementary Table 1). The 3-day menu, included three meals/day, had identical food items (same proportions but with adjusted quantities to meet individual EER and the protocol), meal times and daily distribution of energy, between groups (Supplementary Table 2). Total energy from carbohydrate, fat and protein was 50%, 31% and 19%, respectively (Supplementary Information 2). Home deliveries or participant collection from the laboratory occurred weekly. Participants were free-living but instructed to follow the prescribed meal plan. Submission to the experimenter, in real time, of a photograph of each meal was required prior to eating; leftovers, or additional food eaten, were logged. Interstitial glucose was monitored by a Medtronic MiniMed iPro™2 (Northridge, CA, USA) CGM system (Days 8–13) with four calibration capillary finger-prick glucose measures per day (ACCU-CHEK^®^ Performa Blood Glucose Meter, Roche, Germany) and the pattern of glucose excursions was used to verify the meal times.

### Laboratory-visit protocol pre- and post-intervention

Fasting appetite and metabolic measurements were made at ~8:30 a.m., following an overnight fast, and for 3 h, following a standardised liquid breakfast (meal tolerance test, MTT), consumed at ~9:30 a.m. (Ensure^®^ Compact, Abbott Nutritional Ireland, 10 kJ/mL, vanilla flavour; 42 kJ/kg BW).

Baseline resting energy expenditure (REE) and respiratory exchange ratio (RER) were measured using a Quark CPET open-circuit metabolic cart (Cosmed, Quark CPFT, Rome, Italy) (mid 15 min of each 20 min measurement) and the abbreviated Weir equation [33]. Postprandial REE was measured (20 min in every hour) for 3 h after the MTT, enabling calculation of diet-induced thermogenesis (DIT).

Two baseline arterialised venous blood samples were obtained from a retrograde 20-G cannula (Ohmeda, Sweden) placed in a dorsal hand vein, using a warm-air box (55 °C) [34], for mean fasting whole-blood glucose, serum insulin, plasma FFA, serum TAG, total cholesterol, HDL-cholesterol and LDL-cholesterol. Further arterialised samples were obtained every 10 min for glucose and every 20 min for insulin, FFA and TAG concentrations, for 3 h after the MTT. The HOMA-IR was used to quantify fasting insulin sensitivity and pancreatic β-cell function [35] and the Matsuda index, over 180 min, for evaluation of insulin sensitivity after the MTT, reflecting hepatic and peripheral tissue postprandial sensitivity to insulin [36].

Subjective appetite ratings were obtained at baseline and every 20 min for 3 h after the MTT and every 20 min for an hour after the ad libitum test meal using visual analogue scales [37]. The composite appetite score (CAS) was calculated as follows [38]:

$${\rm{CAS}} = [{\rm{hunger}} + {\rm{desire}}\,{\rm{to}}\,{\rm{eat}} + {\rm{prospective}}\,{\rm{food}}\,{\rm{consumption}} +\,\, (100 - {\rm{fullness}}) + (100 - {\rm{satisfaction}})]/5$$

A higher CAS value is indicative of greater motivation to eat or less feeling of satiety.

Three hours after the MTT, a ~430 g portion of a pasta based test meal was served at ≥82 °C, as a standardised measure of ad libitum food intake [39] (composition: 620 kJ/100 g with 49.8% carbohydrate, 15.4% protein and 34.8% fat) to participants instructed to eat until they felt ‘comfortably full’. The bowl was repeatedly topped up, when about two thirds had been consumed. A final blood sample was taken 1 h after the ingestion of the ad libitum test meal, for determination of glucose concentration.

Immediately after each sampling, whole-blood glucose was analysed with a Yellow Springs^TM^ glucose and lactate analyser (YSI 2300 STAT PLUS, Yellow Springs Inc., Ohio, USA) and the remaining sample was used to obtain serum or plasma. Serum insulin was determined by the human radioimmunoassay kit (HI-14K; Merck Millipore, MA, USA) [40], plasma FFA concentrations using a kit (NEFA-HR-2, Wako, Germany) run on a photometric auto-analyser (ABX Pentra 400, Horiba Ltd., France) as were serum total cholesterol, HDL-cholesterol, LDL-cholesterol and TAG concentrations with reagents from Horiba Medical (France).

### Statistical analyses

Prism 8 software (GraphPad Software Inc., CA, USA) was used for data entry and analyses (statistical significance accepted as *P* < 0.05). Values in the text, figures and tables are mean and SD unless otherwise stated. Data were tested for normality using the D’Agostino–Pearson normality test. iAUC and decremental area under the curve (dAUC) of the postprandial measurements were calculated using the trapezoid rule. Unpaired Student’s *t* tests compared single data points between the two groups. Two-factor Mixed Model ANOVA (between groups factor: treatment, 5:2 IER vs. CER; within groups factor: sampling time; pre vs. post each 2-week energy restricted intervention) evaluated intervention effects; when significant treatment, time or treatment × time interaction effects were observed, post hoc comparisons (where relevant) were explored using paired or unpaired Student’s *t* test. Effect size was estimated as partial eta squared (*η*^2^_*p*_), which is the standard method giving the proportion of variance associated with two-way ANOVA analysis.

In response to limited previous studies on postprandial responses to 5:2 IER or 5:2 intermittent fasting, the power analysis was based on an intermittent fasting study using glucose disposal as an index of postprandial insulin sensitivity [41]. The statistical power analysis indicated that eight participants were required per group to detect a 16% improvement in postprandial whole-body insulin sensitivity with a power of 80% at a significance level of *P* < 0.05.

The primary outcome of this study was the iAUC for insulin for 3 h after a standardised liquid breakfast. Secondary outcomes were changes in postprandial glucose, FFA, TAG, CAS and Matsuda index, energy intake of the ad libitum test meal and whole-day glycaemic profiles for the average of 6 consecutive days. Exploratory outcomes included fasting blood glucose, fasting ratings of subjective appetite, fasting REE, DIT and fasting and postprandial RER responses.

## Results

### Participant characteristics

Eight participants per group completed the study (CONSORT diagram in Supplementary Fig. 2) with characteristics at screening, detailed in Supplementary Table 3, showing matching between groups for age, sex and anthropometrics.

5:2 IER and CER were equally effective for weight loss (mean weight change: −2.5 (SD 1.0) kg 5:2 IER, −2.3 (SD 0.7) kg CER after 2-week intervention; −1.3 (SD 1.2) kg 5:2 IER, −1.1 (SD 0.5) kg CER after 1-week intervention) and had comparable reductions in waist and hip circumferences. There was a significant treatment-by-time interaction for fasting HR (*P* < 0.05, two-way ANOVA). Both groups had similar habitual daily steps, which were comparable to during the intervention, as was fasting BP (Table 1).

**Table 1:** Anthropometric, physiological characteristics and blood measurements before (pre) and after (post) each intervention^a^.

	5:2 IER (*n* = 8)		CER (*n* = 8)		5:2 IER vs. CER^b^
	Pre	Post	Pre	Post	
	Mean ± SD	Mean ± SD	Mean ± SD	Mean ± SD	
Body weight, kg	63.4 ± 14.0	60.9 ± 13.2	66.1 ± 11.2	63.9 ± 11.0	NS
Waist, cm	71.1 ± 8.7	68.7 ± 8.1	75.2 ± 6.7	70.8 ± 5.2	NS
Hip, cm	96.3 ± 4.7	93.8 ± 6.1	96.7 ± 7.2	93.6 ± 7.1	NS
Heart rate, BPM	68 ± 6	62 ± 6	60 ± 8	60 ± 7	<0.05
DBP, mmHg	65 ± 2	65 ± 5	63 ± 8	63 ± 5	NS
SBP, mmHg	110 ± 11	108 ± 11	104 ± 13	103 ± 10	NS
Fasting glucose, mmol/L	4.39 ± 0.28	4.07 ± 0.23	4.42 ± 0.16	4.43 ± 0.17	<0.05
Fasting insulin, mIU/L	9.40 ± 3.06	7.78 ± 2.87	7.98 ± 1.54	6.43 ± 1.78	NS
Fasting FFA, mmol/L	0.46 ± 0.20	0.43 ± 0.10	0.43 ± 0.16	0.45 ± 0.11	NS
Fasting TAG, mmol/L	0.80 ± 0.37	0.64 ± 0.28	0.68 ± 0.26	0.60 ± 0.16	NS
Fasting total cholesterol, mmol/L	3.64 ± 0.37	3.35 ± 0.45	3.76 ± 0.63	3.62 ± 0.54	NS
Fasting LDL-C, mmol/L	2.03 ± 0.23	1.87 ± 0.41	2.15 ± 0.42	2.06 ± 0.43	NS
Fasting HDL-C, mmol/L	1.17 ± 0.30	1.06 ± 0.25	1.20 ± 0.21	1.16 ± 0.16	NS
Glucose iAUC, mmol/L × 180 min	261 ± 124	334 ± 173	222 ± 76	247 ± 65	NS
Insulin iAUC, mIU/L × 180 min	8633 ± 4016	7419 ± 2983	6270 ± 1721	6330 ± 2436	NS
TAG iAUC, mmol/L × 180 min	62 ± 60	36 ± 22	42 ± 32	29 ± 22	NS
FFA dAUC, mmol/L × 180 min	−54 ± 30	−46 ± 10	−52 ± 22	−53 ± 17	NS
Fasting CAS, mm	83 ± 4	63 ± 14	73 ± 11	83 ± 12	<0.001
CAS dAUC (180 min after the MTT)	−5570 ± 2549	−5853 ± 1592	−5355 ± 2093	−6112 ± 2241	NS
CAS dAUC (60 min after the ad libitum test meal)	−4944 ± 1248	−3850 ± 1546	−4472 ± 1079	−4580 ± 1274	NS
Daily step count (habitual levels and levels during interventions)	9650 ± 2771	9780 ± 3227	9725 ± 2444	9481 ± 1869	NS

*5:2 IER* 5:2 intermittent energy restriction, *CER* continuous energy restriction, *NS* not statistically significant, *DBP* diastolic blood pressure, *SBP* systolic blood pressure, *iAUC* incremental area under the curve, *dAUC* decremental area under the curve, *CAS* composite appetite score, *MTT* meal tolerance test (a standardised liquid breakfast).

^a^Blood measurements include fasting blood measurements, postprandial incremental area under the curve (Glucose, Insulin and TAG) and decremental area under the curve (FFA) for blood measurements over 180 min after consumption of the liquid breakfast.

^b^Two-way ANOVA, treatment-by-time interaction.

### Postprandial serum insulin responses

**Fig. 1:**
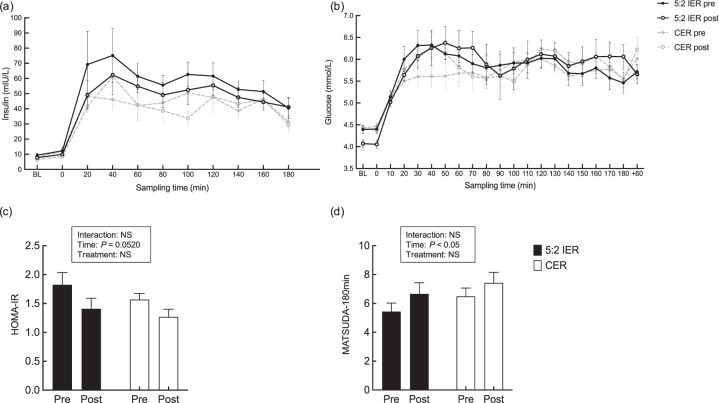
Metabolic response to the liquid breakfast and ad libitum meal. Serum insulin (**a**), whole-blood glucose (**b**) following the liquid breakfast (both parameters) and the ad libitum meal (glucose only); insulin sensitivity assessed using the HOMA-IR (**c**) and Matsuda index (**d**). Pre- pre-intervention measurement, Post- post-intervention measurement, 5:2 IER 5:2 intermittent energy restriction, CER continuous energy restriction, FFA free fatty acid, TAG triglycerides, HOMA-IR homoeostasis model assessment of insulin resistance. **a**–**d** Values are means with their standard errors. **a**–**d** *n* = 8 per group.

Serum insulin rapidly increased and peaked after 20–40 min, before steadily declining, but did not return to fasting levels within 180 min (Fig. 1a). There was no significant treatment-by-time interactions between groups (*P* = 0.31, two-way ANOVA, *η*^2^_*p*_ = 0.01) or main effects of treatment or time in iAUC for insulin over 180 min postprandially (Table 1).

### Fasting and postprandial whole-blood glucose responses

Whole-blood glucose concentrations increased rapidly after the MTT and remained above fasting levels for 180 min (Fig. 1b). There were no significant treatment-by-time interactions or main effects of treatment or time in iAUC for whole-blood glucose (Table 1). However, as an exploratory finding, there was a significant treatment-by-time interaction between the 5:2 IER and CER regimens for fasting blood glucose (*P* = 0.02, two-way ANOVA, *η*^2^_*p*_ = 0.14); with post-intervention values significantly lower after 5:2 IER than CER (*P* < 0.01) (Table 1).

### Whole-body insulin sensitivity

There was no significant treatment-by-time interaction between groups in HOMA-IR (Fig. 1c); but there was a tendency to a decrease in both groups over the 2-week intervention period (*P* = 0.052, main effect of time, two-way ANOVA, *η*^2^_*p*_ = 0.15). Similarly, no significant treatment-by-time interaction between groups was detected in the Matsuda index (over 180 min) (Fig. 1d). However, the Matsuda index increased in both groups over the 2-week interventions (*P* = 0.02, main effect of time, two-way ANOVA, *η*^2^_*p*_ = 0.08), indicating improvement in whole-body insulin sensitivity.

### Postprandial FFA and TAG responses

A reduction in plasma FFA concentrations occurred in both groups, following the MTT (Supplementary Fig. 3a and Table 1). TAG concentrations steadily increased after meal consumption (Supplementary Fig. 3b and Table 1). There were no significant treatment-by-time interactions or main effects of treatment or time between groups in AUCs for plasma FFA and serum TAG.

### Fasting and postprandial CAS responses

**Fig. 2:**
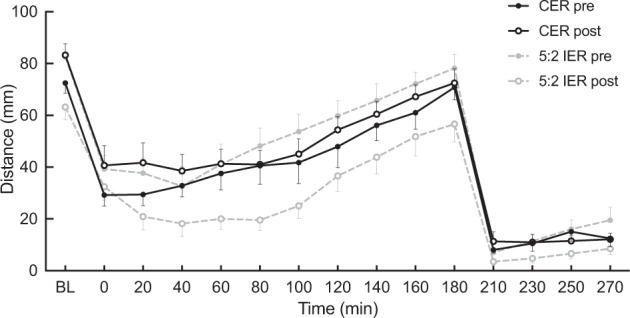
Mean composite appetite score (CAS). 5:2 IER pre/post-5:2 intermittent energy restriction pre-intervention/post-intervention measurement, CER pre/post continuous energy restriction pre-intervention/post-intervention measurement. Values are means with their standard errors. *n* = 8 per group.

There were no significant treatment-by-time interactions in CAS dAUCs over 3 h after the MTT or 1 h after the ad libitum test meal between the two groups (Fig. 2 and Table 1). Interestingly, fasting CAS showed significant treatment-by-time interaction between the two groups (*P* < 0.001) (Table 1) with fasting CAS decreasing after the 5:2 IER and increasing after the CER treatment.

### Energy intake of the ad libitum test meal

There was no treatment-by-time interaction or a main effect of treatment or time for ad libitum test meal energy intake: 2253 (SD 956), 1798 (SD 704), 1898 (SD 462) and 1883 (SD 857) kJ in the pre- and post-5:2 IER and the pre- and post-CER visits, respectively. No interaction or main effects of eating duration or eating speed were identified between groups.

### Free-living continuous glucose monitoring

**Fig. 3:**
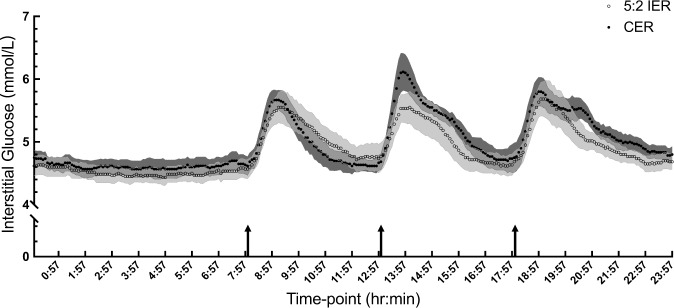
Whole-day glycaemic profiles calculated as mean interstitial glucose concentrations of 6 consecutive days (Days 8–13) for six participants of the 5:2 IER (○) and CER (●). 5:2 IER 5:2 intermittent energy restriction, CER continuous energy restriction. Values are means with their standard errors. SEM for the 5:2 IER is the light shading and for the CER is the dark shading. *n* = 6 per group. Black arrows at 08:00, 13:00 and 18:00 indicate when the breakfast, lunch and dinner started, respectively.

Whole-day glycaemic profiles, with each timepoint representing the mean of 6 consecutive days (Days 8–13) per participant, averaged over the participants, by treatment, are presented in Fig. 3 (restricted intake on Days 8 and 11 for the 5:2 IER group). Shaded error bands (SEM) indicate the inter-individual variation for the mean of 6 days. Both groups exhibit sharp elevations in interstitial glucose concentrations following consumption of the three main meals, peaking after ~1 h and returning to within the fasting range before the next meal. Responses were similar between the two groups (Table 2).

**Table 2:** Indices derived from 24 h continuous glucose monitoring during Days 8–13 of both interventions.

	Full 24 h			Day hours (0700–2359)			Night hour (2400–0659)		
	5:2 IER (*n* = 6)	CER (*n* = 6)	*P*^a^	5:2 IER (*n* = 6)	CER (*n* = 6)	*P*^a^	5:2 IER (*n* = 6)	CER (*n* = 6)	*P*^a^
Valid days	Days 8–13	Days 8–13		Days 8–13	Days 8–13		Days 8–13	Days 8–13	
	Mean ± SD	Mean ± SD		Mean ± SD	Mean ± SD		Mean ± SD	Mean ± SD	
Mean glucose, mmol/L	4.8 ± 0.3	5.0 ± 0.3	NS	5.0 ± 0.4	5.1 ± 0.3	NS	4.5 ± 0.3	4.6 ± 0.3	NS
Maximum, mmol/L	6.0 ± 0.6	6.3 ± 0.6	NS	6.0 ± 0.6	6.3 ± 0.6	NS	4.7 ± 0.4	4.8 ± 0.3	NS
Minimum, mmol/L	4.3 ± 0.4	4.4 ± 0.4	NS	4.4 ± 0.4	4.4 ± 0.4	NS	4.4 ± 0.3	4.5 ± 0.3	NS
SD	0.4 ± 0.2	0.5 ± 0.1	NS	0.4 ± 0.2	0.5 ± 0.2	NS	0.1 ± 0.0	0.1 ± 0.0	NS
% CV	8.4 ± 3.2	9.4 ± 3.1	NS	8.0 ± 3.0	9.1 ± 3.8	NS	1.6 ± 0.6	1.6 ± 0.6	NS

*5:2 IER* 5:2 intermittent energy restriction, *CER* continuous energy restriction, *NS* not statistically significant, *% CV* percentage coefficient of variation.

^a^Unpaired Student’s *t* test.

### Resting energy expenditure (indirect calorimetry data)

There was no significant difference between groups in fasting REE although a significant main effect of time was observed (*P* < 0.05, two-way ANOVA), which showed a reduction by ~0.26 kJ/min in 5:2 IER and by 0.08 kJ/min in CER (Supplementary Fig. 4a). DIT (iAUC for the entire postprandial REE response) showed no significant interaction or treatment or time effect between diet groups (two-way ANOVA) (Supplementary Fig. 4b); nor did either fasting or postprandial RER responses differ between groups (Supplementary Fig. 4c).

## Discussion

The present study demonstrates that under free-living conditions, 2 weeks of 5:2 IER fails to improve postprandial insulin iAUC response (primary outcome) compared with CER in healthy young men and women. Nor were postprandial glucose, FFA, TAG, CAS or energy intake at the ad libitum test meal (secondary outcomes) different. 5:2 IER resulted in a decrease in fasting glucose and fasting CAS compared with CER (exploratory outcome); concurrent weight loss was comparable (3–4% of original BW).

The absence of differences in postprandial insulinaemic response (with small effect size) and postprandial glycaemic, FFA and TAG responses to MTT suggests that metabolic differences (e.g., fasting insulin and insulin resistance) observed between these diets in previous, longer trials [16, 23] cannot be explained by changes in postprandial responses. However, lower fasting glucose following 5:2 IER in our current study (with large effect size) has been observed previously [42]. Further investigation is warranted, of improved hepatic insulin sensitivity, to elucidate the mechanisms underlying the observed differences in fasting blood glucose levels.

Whole-body postprandial insulin sensitivity (Matsuda index) improved equally (with intermediate effect size), along with a strong tendency for increased fasting insulin sensitivity (HOMA-IR) (with large effect size) in both groups, which were probably attributable to equivalent weight loss. Greater reductions in fasting insulin concentration and insulin resistance (HOMA-IR) were reported following 5:2 IER compared with CER in two similar studies [16, 23], however, 2-day energy restriction was on consecutive days and participants had overweight or obesity. Importantly, in our and the previous studies, assessments were made at least 3 days after the last energy-restricted day with 5:2 IER; favourable metabolic effects of 5:2 IER appear not to be due to an acute carry over effect of greater negative energy balance during the last energy-restricted day with 5:2 IER.

Although no significant differences in postprandial CAS response were observed between interventions, beneficial changes in fasting CAS (with large effect size) and individual subjective appetite and satiety ratings were observed in the 5:2 IER but not CER group. These differences might be expected to support long-term compliance and are in common with a study in which participants had obesity; but in contrast to another considering participants with normal weight [43, 44]. In participants with T2DM and overweight or obesity, both groups reported comparable reductions in appetite and increased feelings of fullness and satisfaction [18]. However, participants were taking hypoglycaemic drugs, potentially impacting on glycaemia and appetite.

Hyperphagia was not seen in either group at the ad libitum lunch (energy intake: 20% of their EER), but decreased fasting CAS post-5:2 IER was not associated with a significantly decreased energy intake at this meal; although a numerical decrease was observed with post-5:2 IER intervention. An accumulatively greater difference in total energy intake might occur across all meals of the day. However, subjective and objective measures of appetite may not always align [45], especially in relatively short-term interventions.

The present study extends the investigations of intermittent fasting [41, 46] and other metabolic challenges [39, 47] by considering both fasting and postprandial state in young, normal-weight participants in whom effects on metabolism and appetite are not confounded by the metabolic abnormalities associated with excess BW. Importantly when studying meal pattern and metabolism, and in contrast to previous studies, food was provided over the intervention period and compliance monitored by CGM. Three major excursions in daily glucose profiles were seen in the CGM, coinciding with prescribed meal times, reflecting good dietary adherence, facilitated by food provision. This counters the potential limitation of the participants being free-living [48, 49], as did monitoring activity by using step counts. The balanced sex ratio of participants may help to offset any sex bias, hence making this study more representative of the general population. In this study, pre-intervention metabolic measurements may be confounded by the dinner consumed the evening before. A standardised dinner, prior to each measurement day, may help to minimise the effects of diverse dietary characteristics of the dinner on fasting and postprandial substrates metabolism [50]. A longer study period would confirm stability of the effects noted.

This study shows that 5:2 IER is not superior in improving postprandial insulin and glucose responses compared with CER, suggesting that these are not key to metabolic differences in longer-term intervention studies. Interestingly, the 5:2 IER regimen led to potentially beneficial changes in fasting blood glucose and fasting subjective appetite scores, which were independent of weight loss, over a 2-week period. This was consistent with similar studies conducted in participants with overweight or obesity. Further studies are required to establish mechanisms of action and to examine long-term adherence, safety and efficacy of intermittent fasting.

## Supplementary information

Supplementary Information

5to2IER Supplementary tables

Supplementary figure legend

Supplementary Fig. S1.

Supplementary Fig. S2.

Supplementary Fig. S3.

Supplementary Fig. S4.
