# Supplementary material for: Effects of intermittent (5:2) or continuous energy restriction on basal and postprandial metabolism: a randomised study in normal-weight, young participants
Source: Eur J Clin Nutr. 2021 May 26;76(1):65–73. doi: 10.1038/s41430-021-00909-2 (PMC8766278; doi:10.1038/s41430-021-00909-2)
Supplement: Supplementary file 1 — Supplementary Information [file 41430_2021_909_MOESM1_ESM.docx]

**Supplementary Information 1. Exclusion criteria**

Exclusion criteria included: smoking; regularly skipping breakfast; any metabolic, endocrine or cardiovascular abnormalities; taking any medication other than over-the-counter drugs; individuals with high intensity physical training; shift workers; on an energy-restricted diet; high alcohol consumption; women who were pregnant, lactating or with irregular menstrual cycles; self-reported allergy, intolerance or strong dislike of foods or drinks to be offered during the intervention; Beck Depression Inventory or Eating Attitudes Test scores providing evidence of depression (BDI score > 10) or eating disorder (EAT-26 score > 20).

**Supplementary Information 2. Menu design**

Each menu provided 3 meals/day to avoid a potential confounding effect caused by different meal frequencies in the two interventions. Three meals/day (and no snacks) was selected to make each eating incident acceptable, with respect to energy content, on the most restricted days. Three meals with a macronutrient distribution of ~50% energy from carbohydrates, ~31% from fat and ~19% from protein were prescribed. This maintained the same absolute amount of protein as would be provided by a non-restricted diet in which 15% of energy was from protein. The distribution of energy across the day was 25% of daily energy requirements from breakfast, 37.5% from lunch and 37.5% from evening meal. To avoid potential confounding by altering the meal time, instructions were given to consume breakfast between 8 am - 8:30 am; lunch between 1 pm - 1:30 pm and evening meal between 6 pm - 6:30 pm. Food composition was matched on a “meal-by-meal” basis in and between both groups to eliminate any confounding effects caused by macronutrient difference. Food items were identical between groups across the intervention to eliminate any confounding effects caused by textural and visual differences. The menu was designed so that the energy provided per meal could be proportionally increased and decreased depending on the intervention requirements and was informed by likely availability of cooking facilities and to optimising food safety. Participants were also asked to refrain from alcohol and caffeine consumption.
