# Supplementary material for: Effects of intermittent (5:2) or continuous energy restriction on basal and postprandial metabolism: a randomised study in normal-weight, young participants
Source: Eur J Clin Nutr. 2021 May 26;76(1):65–73. doi: 10.1038/s41430-021-00909-2 (PMC8766278; doi:10.1038/s41430-021-00909-2)
Supplement: Supplementary file 2 — 5to2IER Supplementary tables [file 41430_2021_909_MOESM2_ESM.docx]

**Supplementary Table 1.** The diet pattern throughout the interventions in 5:2 IER and CER groups

| Day | 1 | 2 | 3 | 4 | 5 | 6 | 7 | 8 | 9 | 10 | 11 | 12 | 13 | 14 |
| --- | --- | --- | --- | --- | --- | --- | --- | --- | --- | --- | --- | --- | --- | --- |
| Day of menu cycle | A | B | C | A | B | C | A | B | C | A | B | C | A | B |
| **5:2 IER**  **Semi fasting day**  **(30% EER)** | 30% EER |  |  | 30% EER |  |  |  | 30% EER |  |  | 30% EER |  |  |  |
| **5:2 IER**  **Non fasting day**  **(100% EER)** |  | 100% EER | 100% EER |  | 100% EER | 100% EER | 100% EER |  | 100% EER | 100% EER |  | 100% EER | 100% EER | 100% EER |
| **CER**  **(80% EER)** | 80% EER | 80% EER | 80% EER | 80% EER | 80% EER | 80% EER | 80% EER | 80% EER | 80% EER | 80% EER | 80% EER | 80% EER | 80% EER | 80% EER |

*5:2 IER* 5:2 intermittent energy restriction, *CER* continuous energy restriction, *EER* estimated energy requirements

**Supplementary Table 2.** Examples of menus A,B,C throughout the interventions

(For **8368 kJ or 2000 kcal** of total intake)

| **Menu A** | **Menu B** | **Menu C** |
| --- | --- | --- |
| **Breakfast** | **Breakfast** | **Breakfast** |
| Cheerios Cereal, 30g  British Semi Skimmed Milk, 200g  Pancakes, 56g  Peanut Butter Smooth, 14g  Cashew Kernels, 10g | Cheerios Cereal, 30g  British Semi Skimmed Milk, 200g  Pancakes, 56g  Peanut Butter Smooth, 14g  Cashew Kernels, 10g | Cheerios Cereal, 30g  British Semi Skimmed Milk, 200g  Pancakes, 56g  Peanut Butter Smooth, 14g  Cashew Kernels, 10g |
| **Lunch** | **Lunch** | **Lunch** |
| Slow Cooked Spaghetti Bolognese, 400g  Gala Apples, 225g  No Added Sugar 0% Fat Peach Yogurt, 125g | Chicken & Bacon Pasta Bake, 400g  Easy Peelers, 70g  Chocolate Chip Muffins, 30g  No Added Sugar 0% Fat Strawberry Yogurt, 125g | Classic Shepherd's Pie, 525g  Chocolate Chip Muffins, 30g  Gala Apples, 130g  No Added Sugar 0% Fat Peach Yogurt, 250g |
| **Dinner** | **Dinner** | **Dinner** |
| Mixed Vegetables, 220g  Indian Chicken Tikka Masala, 180g  Rice Egg Fried, 197g  Easy Peelers, 110g | Mixed Vegetables, 178g  Chilli Con Carne, 450g  Chocolate Chip Muffins, 50g  Easy Peelers, 58g | Mixed Vegetables, 115g  Spaghetti Carbonara, 400g  Chocolate Chip Muffins, 30g  Easy Peelers, 55g |

(For **2510 kJ** **or 600 kcal** of total intake, with 70% energy restriction)

| **Menu A** | **Menu B** | **Menu C** |
| --- | --- | --- |
| **Breakfast** | **Breakfast** | **Breakfast** |
| Cheerios Cereal, 9g  British Semi Skimmed Milk, 60g  Pancakes, 16.7g  Peanut Butter Smooth, 4.2g  Cashew Kernels, 3g | Cheerios Cereal, 9g  British Semi Skimmed Milk, 60g  Pancakes, 16.7g  Peanut Butter Smooth, 4.2g  Cashew Kernels, 3g | Cheerios Cereal, 9g  British Semi Skimmed Milk, 60g  Pancakes, 16.7g  Peanut Butter Smooth, 4.2g  Cashew Kernels, 3g |
| **Lunch** | **Lunch** | **Lunch** |
| Slow Cooked Spaghetti Bolognese, 120g  Gala Apples, 67.5g  No Added Sugar 0% Fat Peach Yogurt, 37.5g | Chicken & Bacon Pasta Bake, 120g  Easy Peelers, 21g  Chocolate Chip Muffins, 9g  No Added Sugar 0% Fat Strawberry Yogurt, 37.5g | Classic Shepherd's Pie, 157.5g  Chocolate Chip Muffins, 9g  Gala Apples, 39g  No Added Sugar 0% Fat Peach Yogurt, 75g |
| **Dinner** | **Dinner** | **Dinner** |
| Mixed Vegetables, 66g  Indian Chicken Tikka Masala, 54g  Rice Egg Fried, 59g  Easy Peelers, 33g | Mixed Vegetables, 53.4g  Chilli Con Carne, 135g  Chocolate Chip Muffins, 15g  Easy Peelers, 17.4g | Mixed Vegetables, 34.5g  Spaghetti Carbonara, 120g  Chocolate Chip Muffins, 9g  Easy Peelers, 16.5g |

**Supplementary Table 3.** Descriptive characteristics for completers at screening

|  | 5:2 IER (*n* 8) | |  | CER (*n* 8) | |  |
| --- | --- | --- | --- | --- | --- | --- |
|  | Mean | SEM |  | Mean | SEM | 5:2 IER v. CER ^a^ |
| Sex (*n*) |  |  |  |  |  |  |
| Male | 4 | |  | 4 | |  |
| Female | 4 | |  | 4 | |  |
| Age (years) | 23 | 1 |  | 26 | 2 | NS |
| Body weight (kg) | 63.1 | 4.9 |  | 66.1 | 4.0 | NS |
| BMI (kg/m^2^) | 21.7 | 0.8 |  | 22.7 | 0.6 | NS |
| Body fat (%) ^b^ | 20.2 | 2.0 |  | 22.1 | 2.7 | NS |

*NS* not statistically significant

^a^ Unpaired student’s t-test

^b^ Estimated using skinfold thickness values
