# Supplementary material for: Effects of intermittent (5:2) or continuous energy restriction on basal and postprandial metabolism: a randomised study in normal-weight, young participants
Source: Eur J Clin Nutr. 2021 May 26;76(1):65–73. doi: 10.1038/s41430-021-00909-2 (PMC8766278; doi:10.1038/s41430-021-00909-2)
Supplement: Supplementary file 3 — Supplementary figure legend [file 41430_2021_909_MOESM3_ESM.docx]

**Supplementary Fig. S1.** Schematic diagram of experimental protocol.

**Supplementary Fig. S2.** CONSORT (Consolidated Standards of Reporting Trials) diagram. CGM, continuous glucose monitoring.

**Supplementary Fig. S3.** Plasma FFA (a) and serum TAG (b) following the liquid breakfast. Pre, pre-intervention measurement; Post, post-intervention measurement; 5:2 IER, 5:2 intermittent energy restriction; CER, continuous energy restriction; FFA, free fatty acid; TAG, triglycerides. a,b: values are means with their standard errors. a,b: *n*=8 per group.

**Supplementary Fig. S4.** (a,b,c) Indirect calorimetry measurements. REE, resting energy expenditure; DIT, diet induced thermogenesis; DIT was calculated by the incremental area under the curve for the resting energy expenditure above the fasting value, using trapezoidal method. Pre, pre-intervention measurement; Post, post-intervention measurement; 5:2 IER, 5:2 intermittent energy restriction; CER, continuous energy restriction. a,b,c: values are means with their standard errors. a,b,c: *n*=7 for 5:2 IER and *n*=8 for CER.
