# Supplementary figures and images for: Effects of intermittent (5:2) or continuous energy restriction on basal and postprandial metabolism: a randomised study in normal-weight, young participants
Source: Eur J Clin Nutr. 2021 May 26;76(1):65–73. doi: 10.1038/s41430-021-00909-2 (PMC8766278; doi:10.1038/s41430-021-00909-2)

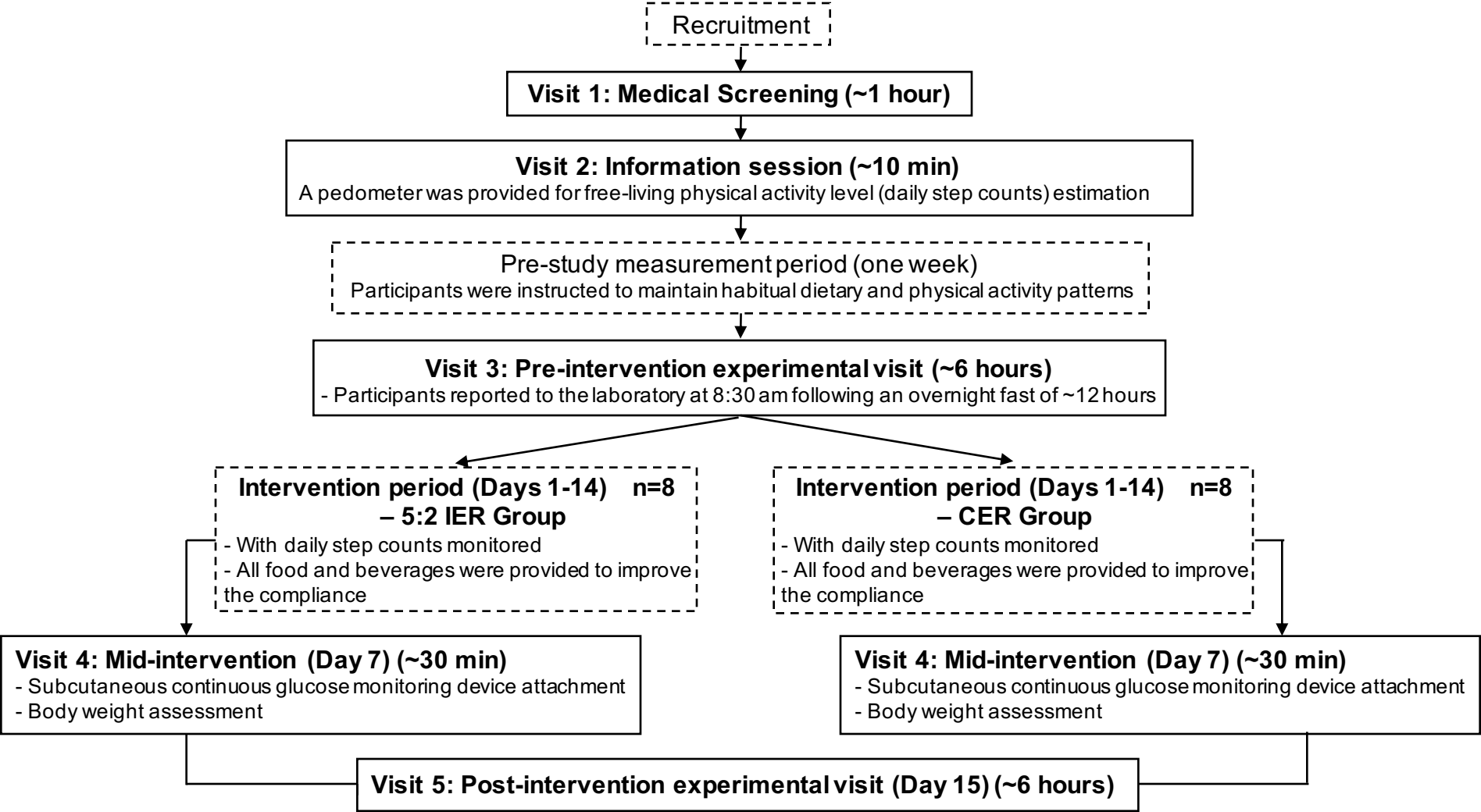

Supplement: Supplementary file 4 — Supplementary Fig. S1. [file 41430_2021_909_MOESM4_ESM.pdf]

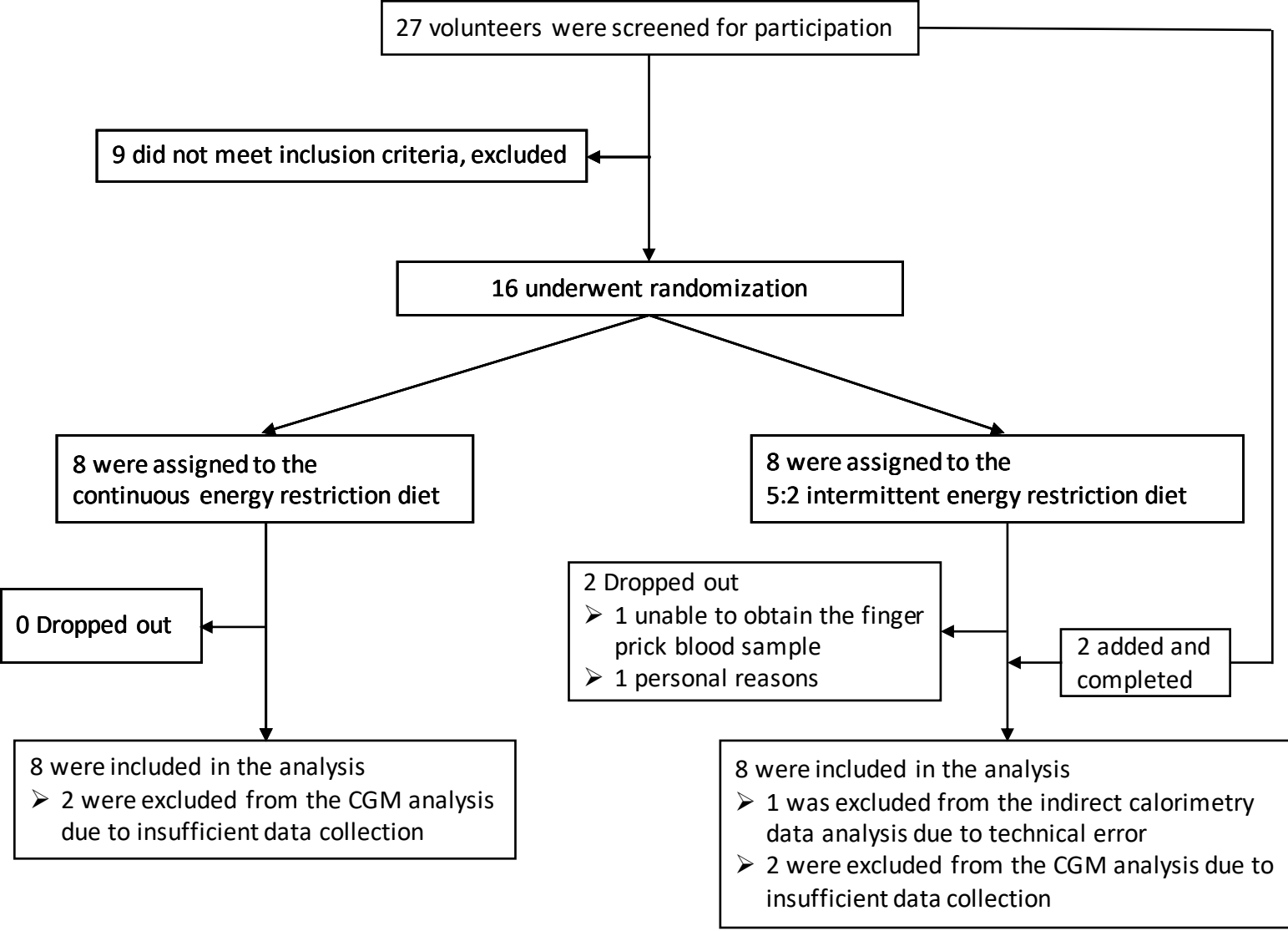

Supplement: Supplementary file 5 — Supplementary Fig. S2. [file 41430_2021_909_MOESM5_ESM.pdf]

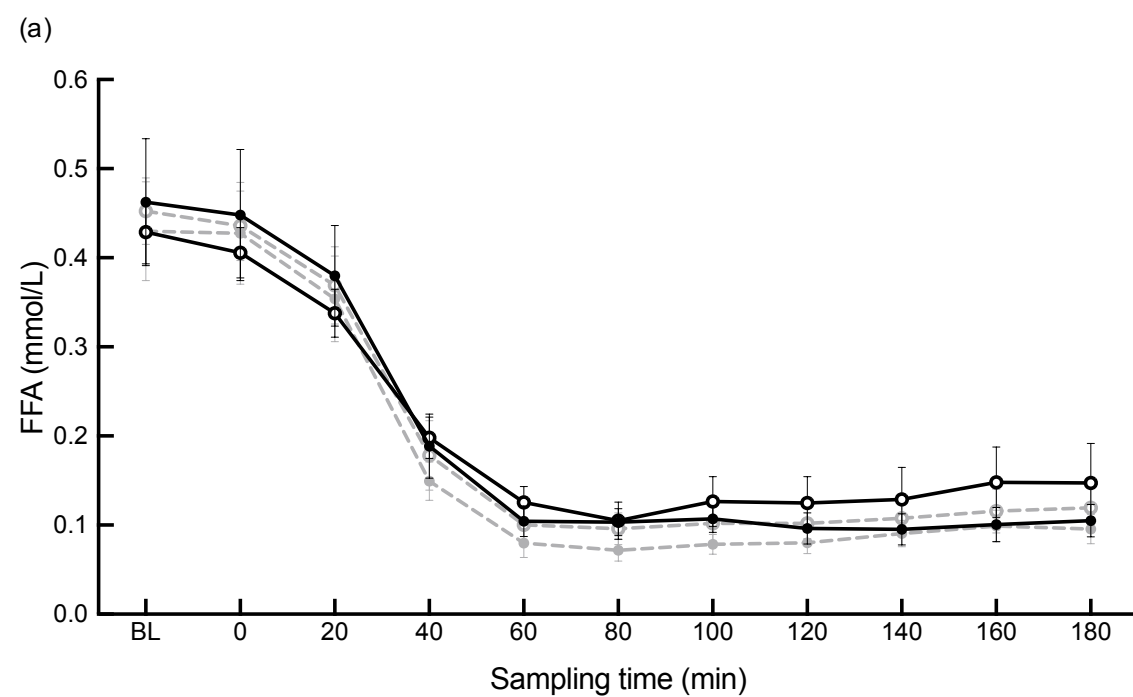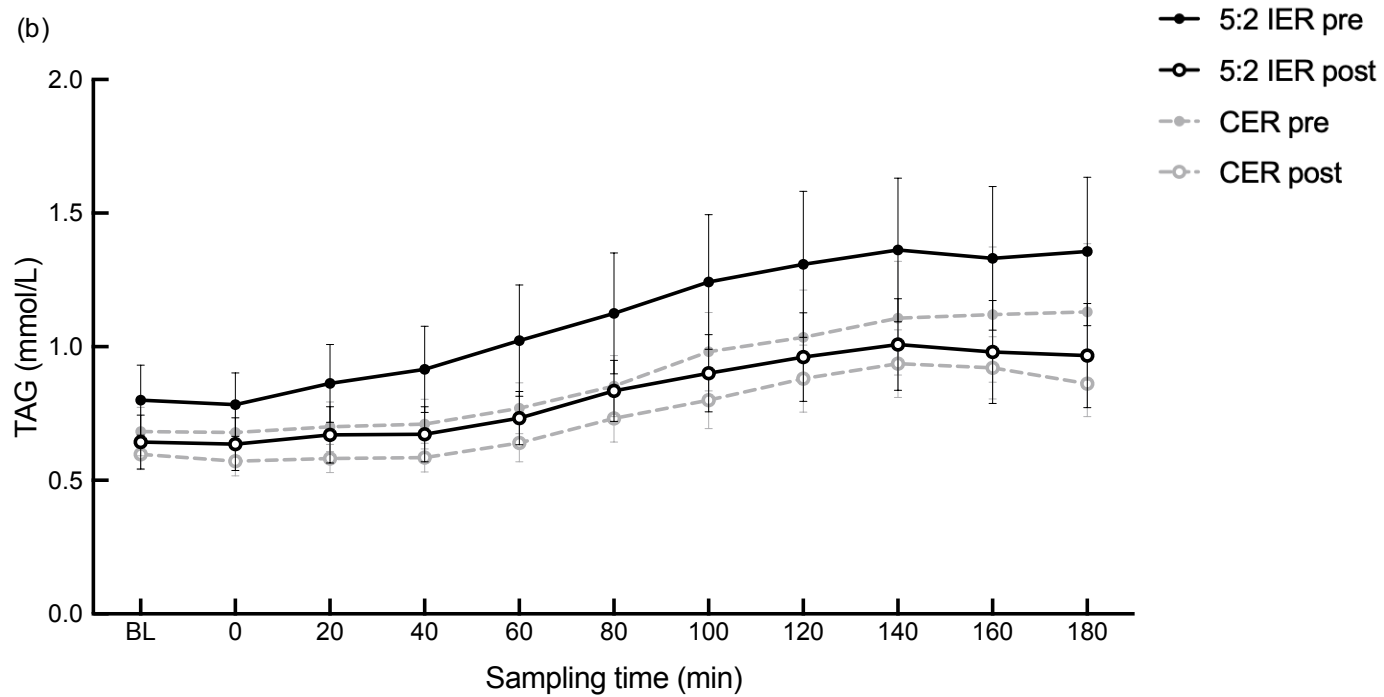

Supplement: Supplementary file 6 — Supplementary Fig. S3. [file 41430_2021_909_MOESM6_ESM.pdf]

(a)

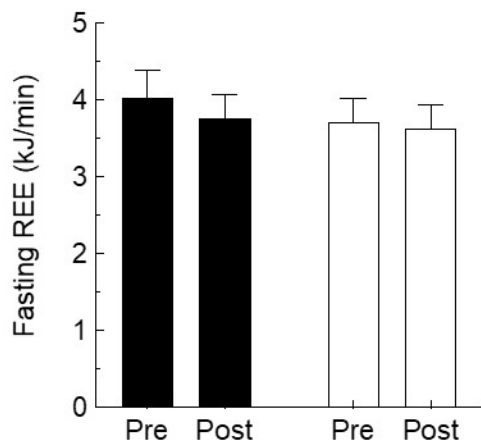

(b)

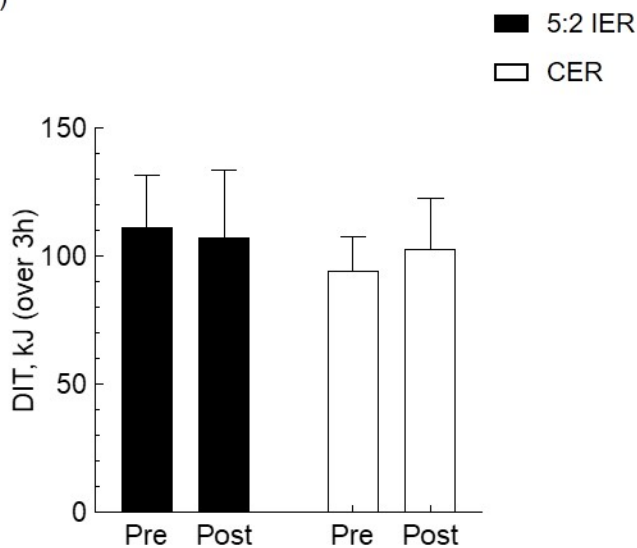

(c)

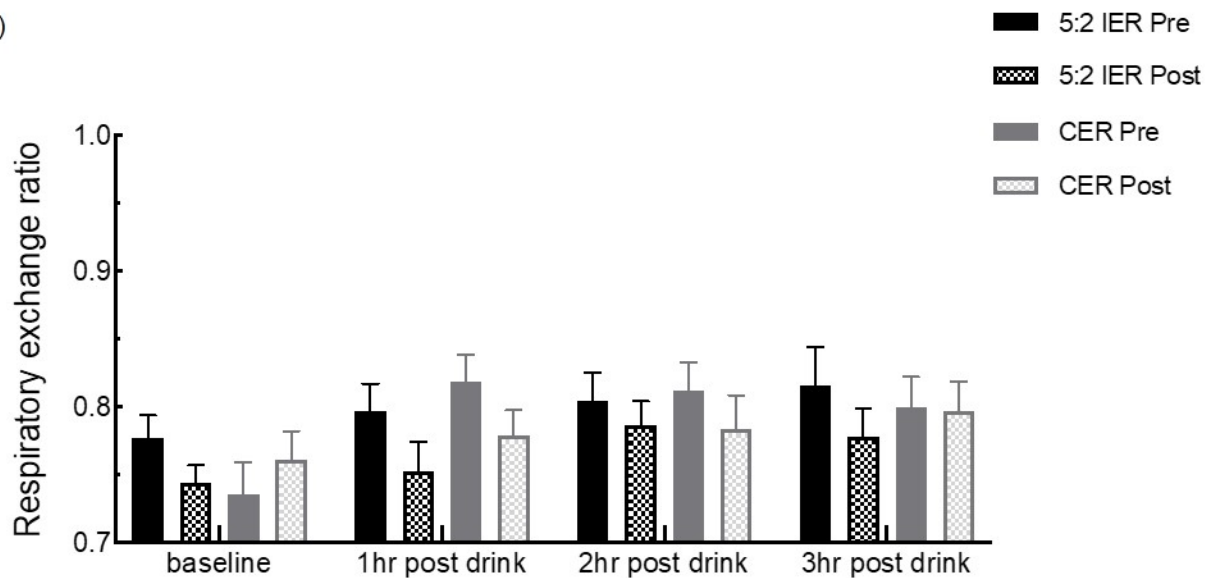

Supplement: Supplementary file 7 — Supplementary Fig. S4. [file 41430_2021_909_MOESM7_ESM.pdf]
